# Deep sedation in lateral position for preterm infants during cerebral magnetic resonance imaging: a pilot study

**DOI:** 10.1186/s44158-024-00216-9

**Published:** 2024-12-18

**Authors:** Fabio Sbaraglia, Simona Gaudino, Eloisa Tiberi, Federica Maiellare, Giorgia Spinazzola, Rossella Garra, Filomena Della Sala, Daniela Maria Micci, Rosellina Russo, Francesca Riitano, Giuseppe Ferrara, Giovanni Vento, Marco Rossi

**Affiliations:** 1 Department of Anesthesia and Intensive Care, Fondazione Policlinico Universitario A. Gemelli IRCCS, Università Cattolica del Sacro Cuore, Rome, Italy; 2 Department of Radiologic and Hematologic Sciences, Fondazione Policlinico Universitario A. Gemelli IRCCS, Università Cattolica del Sacro Cuore, Rome, Italy; 3 Neonatology Unit, Department of Woman and Child Health and Public Health, Fondazione Policlinico Universitario A. Gemelli IRCCS, Rome, Italy

**Keywords:** Sedation, Magnetic resonance, Infant, Premature, Position, Body temperature

## Abstract

**Introduction:**

Respiratory adverse events are common during the sedation of preterm babies, often needing active airway support. During magnetic resonance imaging, this occurrence could extend the acquisition time, with a negative impact on the thermic and metabolic homeostasis. The aim of the study is to verify if lying in a lateral position instead of supine could improve the safe quality of sedation, without worsening the quality of imaging.

**Methods:**

This study was performed as a single-center, prospective study at a university-affiliated tertiary care center. A consultant provided deep sedation with sevoflurane 3–4% delivered by an external mask, in the lateral decubitus position. All patients were evaluated for the incidence of apnea and desaturation, quality of imaging obtained, the timing of imaging acquisition, and thermic and metabolic homeostasis.

**Results:**

We enrolled 23 consecutive preterm babies born < 37 weeks gestational age, candidates for sedation for elective brain magnetic resonance imaging. All patients completed the radiological procedure in 30 min (SD ± 6.39 min) without complications requiring exam interruption. Only one patient (4%) experienced a transient desaturation, while 2 neonates (9%) showed apnea lasting > 20 s. On average, there was a 1 °C decrease in body temperature and full enteral feeding was resumed within 1.5 h. Neuroradiologists rated the quality of the images obtained as high.

**Conclusions:**

Lateral lying seems to be a viable option for sedated preterm babies during magnetic resonance imaging with a low risk of intervention for apnea and a reduced impact on thermic and metabolic homeostasis. Quality of imaging would be preserved maintaining correct scheduling of standard care.

**Trial registration:**

The study was registered at www.ClinicalTrials.gov before enrollment (NCT05776238 on December, 21th 2023).

## Introduction

Respiratory adverse events are a long-standing complication during pediatric and neonatal sedation [1]. Literature suggests an incidence between 4 and 8% of apnea due to immaturity of the respiratory control centers and incomplete maturation of the muscular components of the upper airways [2–4]. They may collapse because of their intrinsic immaturity, which occurs more easily under sedation.

The frequency of apneic episodes is inversely proportional to gestational age (GA) and almost all infants with GA less than 28 weeks are affected. Among risk factors, hypothermia is crucial because thermoregulation is not yet matured in premature infants. Despite improvements in monitoring, provider’s experience, and safety, adverse events occur in more than 8% of children undergoing sedation in magnetic resonance (MR), where the long execution time and need for stillness make it even more challenging [5, 6]. Many centers have proposed a non-pharmacological approach to provide patient immobility, such as the feed and swaddle technique, although the success rate of quality MR images is variable compared with the guaranteed success of pharmacological techniques. [7]. In a recent survey, NICU neonates are managed in deep sedation in over 58% of cases, with more than 55% of them using sevoflurane [8]. To address this lack of evidence, many trials have investigated the roles of different types of sedation, provider experience, and the possibility of conducting MR without sedation [9–12].

However, until now, no trial has considered the effect of lying position. Similar experiences in pediatric anesthesia suggest that airway patency is improved in sedated children when placed in the lateral position, due to the increase in cross-sectional area and total upper airway volume compared to the supine position [13].

The primary aim of this pilot prospective observational study was to determine the incidence of apnea and desaturation in preterm newborns lying in lateral positions during sevoflurane sedation for MR. Secondarily, we evaluated the quality of imaging and MR duration, with an indirect effect on thermal and metabolic homeostasis. Our hypothesis is that patients placed in the lateral position (arbitrarily the left side for the entire sample) would be associated with effective spontaneous breathing, quality of imaging, and homeostasis compared with data suggested by the literature.

## Material and methods

The institutional review board of Fondazione Policlinico Gemelli IRCCS approved this pilot prospective study (Prt. 0005227/23) on February 16th, 2023. The study was conducted at the academic tertiary center, Fondazione Policlinico Gemelli IRCCS, Rome, Italy. All procedures included in the study adhered to the ethical standards of the institutional research committee and were in line with the 1975 Helsinki Declaration and its later amendments. Legal caregivers provided written consent.

We included all premature babies born < 37 weeks GA at our Neonatology Center who underwent elective cranial MR for prematurity follow-up, between March and June 2023. Exclusion criteria included: age at exams > 50 weeks post-conceptual age (PCA), previous treatment with hypothermia for hypoxic-ischemic encephalopathy, positive pressure ventilation within the previous 48 h, upper respiratory tract infections, preexisting cardiac arrhythmias, presence or suspicion of oncological, neuromuscular or metabolic diseases, previous surgery, scalp venous accesses, refusal of informed consent by parents or inability to express it.

### Procedural protocol

Newborns were fasted for at least 6 h after formula feeding or 4 h after breastfeeding. A metal-free cotton footie was used for dressing infants and warm coverings were provided for the transfer to the pre-anesthesia room where temperature is standardized at 21 °C. The clinical chart was reviewed and rectal temperature measurement was obtained before the access to the magnetic field area.

According to the institutional standard procedure, the induction was performed in the MR suite (Datex-Ohmeda Avance, General Electric Co., Boston, USA) with O_2_/N_2_O 50% for 3 min followed by sevoflurane 4% in O_2_/air. Anesthesia maintenance was ensured by sevoflurane 3% via a non-rebreathing mask. A venous needle was inserted, pulse oximetry, electrocardiograph, and capnography (Expression MR 200, Philips HealthCare, Amsterdam, the Netherlands) were monitored and ear defenders were used to reduce noise disturbance. Neonates were then positioned in the left lateral lying, and a gentle strapping was applied to reduce limb movements.

**Fig. 1:**
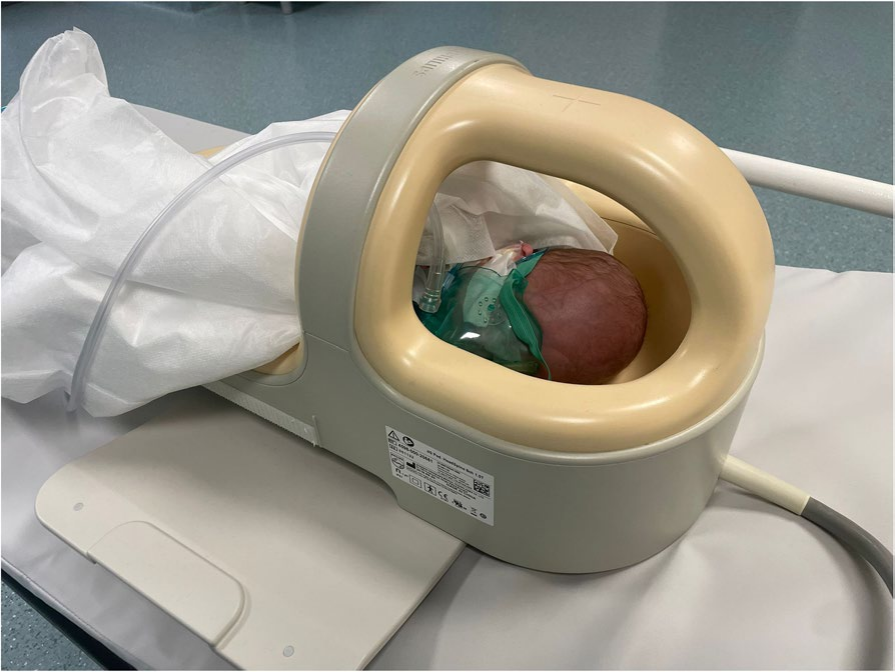
A preterm baby in the final position for MR execution

All images were acquired with a Philips Healthcare Ingenia 1.5 Tesla MR System (Philips Healthcare, Amsterdam, Netherlands), connected to the neonatal dStream Ped NeuroSpine 8ch coil, an open-design 8-element coil for high-resolution pediatric brain and spine imaging (Philips Healthcare, Amsterdam, Netherlands) (Fig. 1).

The MR study protocol for the brain in patients aged 0–6 months was used, with a total duration of approximately 25 min (Table 1). Oxygen saturation, end-tidal CO_2_, respiratory rate, EKG, heart rate, and spontaneous movements were monitored continuously and collected every 5 min.

**Table 1:** Radiologic protocol

Scan	Protocol	
3D T1 (Turbo Field Echo TFE-volumetric)	TR/TE	9.4/4.6 ms
	Matrix size	148 × 119
	In-plane FOV (AP × RL):	146 × 118 mm
	Voxel size:	1 × 1 × 1 mm
	Number of slices	110
	Scan time	2.48 min
Axial T1 (Turbo Spin Echo TSE, SE)	TR/TE	800/15 ms
	Matrix size	160 × 120
	In-plane FOV (AP × RL):	144 × 120 mm
	Voxel size:	0.85 × 1 × 3 mm
	Number of slices	28
	Scan time	4.27 min
Axial T2 (TSE)	TR/TE	8045/140 ms
	Matrix size	220 × 170
	In-plane FOV (AP × RL):	144 × 120 mm
	Voxel size:	0.65 × 0.65 × 3 mm
	Number of slices	28
	Scan time	2.33 min
Axial Diffusion (DWI, ADC)	TR/TE	3499/85 ms
	Matrix size	100 × 86
	In-plane FOV (AP × RL):	158 × 180 mm
	Voxel size:	1.8 × 1.8 × 3 mm
	Number of slices	28
	Scan time	1.35 min
Axial SWI	TR/TE	51/12 ms
	Matrix size	188 × 134
	In-plane FOV (AP × RL)	170 × 141 mm
	Voxel size:	0.9 × 1.06 × 2 mm
	Number of slices	95
	Scan time	2.17 min
Coronal T2	TR/TE	7719/120 ms
	Matrix size	236 × 156
	In-plane FOV (AP × RL)	152 × 111 mm
	Voxel size:	0.65 × 0.65 × 3 mm
	Number of slices	32
	Scan time	3.13 min

*TR/TE* repetition time/echo time, *FOV* field of view, *AP* anterior–posterior, *RL* right-left, *TSE* turbo spin echo, *SE* spin-echo, *DWI* diffusion weighted imaging, *ADC* apparent diffusion coefficient, *SWI* susceptibility weighted imaging

After the MR scan, sevoflurane administration was discontinued and the patient was moved to the nearby post-anesthesia care unit (PACU) for a new rectal temperature measurement and multiparameter monitoring. After 30 min, with stable vital parameters and the resumption of age-appropriate behavior, a neonatologist transferred the neonates to the neonatal intensive care unit (NICU), where normal care of the infant was resumed. Enteral nutrition and ongoing therapies were resumed based on the infant’s clinical stability. Nutrition was provided as it was before MR. Vital sign data and the time required to restore full enteral nutrition were recorded in the medical chart.

### Radiological evaluation

The neuroradiologist evaluated on sight quality of the scan to determine the need for reacquisition. Sequences were then assessed outside the MR suit with both subjective and objective parameters.

Subjective evaluation was performed, following our institutional standard, by two each other blinded neuroradiologists, using a scoring scale of 1–5 (1 = non-diagnostic images, 5 = excellent, completely diagnostic) in five categories (differentiation grey matter-white matter; identification of basal nuclei; visibility of lesions; delineation of lesions; diagnostic utility) according to a 5-point Likert-Scale [14].

**Fig. 2:**
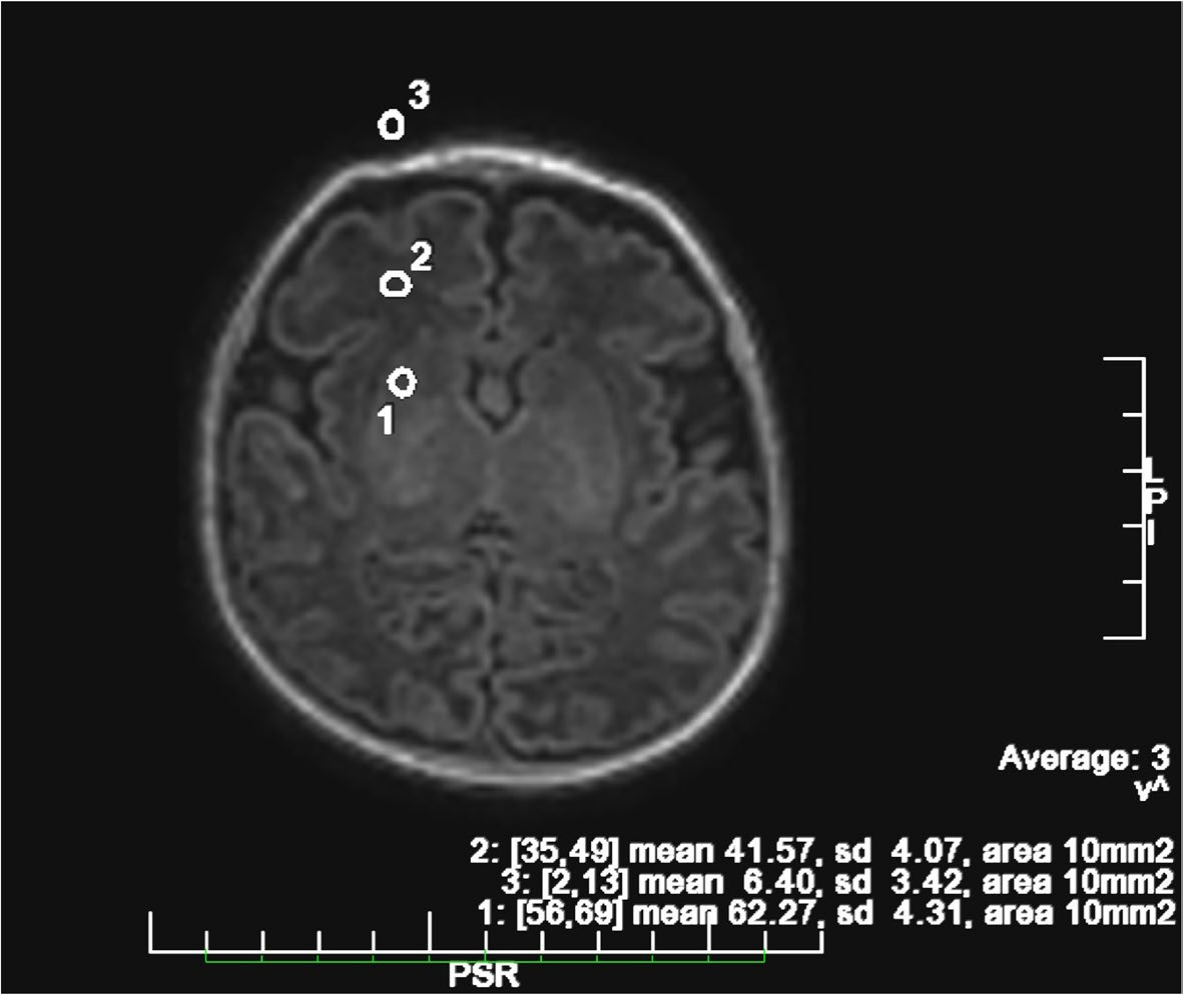
For the objective evaluation, three ROIs were placed in the lenticular nuclei (**1**), normal white matter (**2**), and outside the patient to account for “background noise” (**3**). The values found were then used to calculate the SNRGM, SNRWM, and the CNRGM

Objective evaluation was performed by a blind third radiologist physician [15]. For each MR examination, regions of interest (ROIs) were drawn freehand using the GE Advantage Workstation 4.7 console in the following structures: lenticular nuclei (SGM), normal white matter (SWM), and, in case of lesions, within the lesion itself (SLESION). An ROI of “background noise” (SNOISE) was obtained by drawing the ROI in the airspace surrounding the outside of the skull/brain, avoiding ghosting, aliasing, and eye motion artifact regions (Fig. 2). The average values of SGM, SWM, SLESION were divided by the standard deviation of background noise to obtain the signal-to-noise ratio (SNR).

- SNRGM = SGM / SDNOISE
- SNRWM = SWM / SDNOISE
- SNRLESION = SLESION/ SDNOISE

The contrast-to-noise ratio (CNR) was calculated. For gray matter, it was obtained by the difference in SNR between gray matter and white matter: CNRGM = (SNRGM − SNRWM). In the case of lesions, the lesion’s CNR was defined as the difference in SNR between the lesion and white matter: CNRLESION = (SNRLESION − SNRWM). In the case of lesions, the visibility of the largest lesion will be defined as an absolute value of (SNRLESION − SNRWM/SNRWM) [15, 16].

### Outcome measures

The primary outcome measure was the incidence of respiratory adverse events, associated or not to bradycardia, considered as peripheral saturation < 80%, or apnea > 20 s revealed by end-tidalCO_2_. Secondarily, we evaluate objective and subjective analysis of the quality of imaging and MR scan time (measured from the beginning to the end of the scan). In the absence of a validated quality scale [17, 18], our internal protocol considered an SNR > 10 and a CNR > 5 as excellent.

Change in body temperature was measured through rectal core body temperature (°C) in PACU before and after the exam, while the resumption of full enteral feeding occurred after a complete milk meal, administered after the baby crying.

### Statistical analysis

Given the configuration of this study, as a pilot study due to the lack of literature on the subject, a formal sample size calculation was not performed. Patients were enrolled according to the Birkett & Day Practical Rules on Pilot Studies [19].

All variables included in the study were analyzed using descriptive statistics techniques. In detail, qualitative variables have been described as absolute frequencies and percentages. The Shapiro–Wilk test was used to evaluate the distribution of quantitative variables. The data were expressed as mean and standard deviation (SD), where normally distributed, or as median and interquartile range, otherwise. The acquired data were compared through Student’s *t* test for paired data or Wilcoxon sign rank tests as appropriate, as regards quantitative variables. Qualitative data were evaluated with a chi-square test or two-tailed Fisher’s exact test, as appropriate. A *p* value < 0.05 was considered statistically significant. All analyses were performed using the software Med Calc version 18.6.

## Results

During the study, we enrolled 23 patients and the baseline characteristics are described in Table 2.

**Table 2:** Demographic characteristics

M:F	11:12	
Post-conceptional age (weeks)	31 + 1	
Post-conceptional age at MR (weeks)	37 + 6	
Birth weight (g)	1569	± 747
Weight at MR (g)	2427	± 658

Data are expressed as mean ± standard deviation

All patients completed the radiological procedure without any interruptions.

During the MR, one patient (1/23; 4%) experienced a transient desaturation, while two patients (2/23; 9%) showed apnea lasting > 20 s, without the need for intervention. No patient experienced episodes of bradycardia.

The subjective analysis by both radiologists identified diagnostic utility with the highest score in all patients (Table 3), while objective data (SNRGM, SNWM, and CNR) were 13.94 (IQR 11.4–15.79), 8.46 (IQR 7.04–9.47) and 5.55 (IQR 4.12–6.68) respectively.

**Table 3:** Results

Desaturation (< 80%)	1 of 23
Apnea (> 20 s)	2 of 23
Bradycardia (< 80 bpm)	0/23
GM-WM	5 of 5
Basal nuclei identification	5 of 5
Lesion identification	None
Lesion delimitation	None
Diagnostic utility	5 of 5
SNRGM^a^	13.94 (11.4–15.79)
SNRWM^a^	8.46 (7.04–9.47)
SNRLESION^a^	None
CNRGM	5.55 (4.12–6.68)
CNRLESION	None
Temperature preRM^b^ (°C)	36.8 (36.7–36.9)
Temperature postRM^b^ (°C)	35.8 (35.3–36.2)
Timing full enteral feeding^b^ (h)	1.63 (± 0.83)

^a^SNRGM = SGM SDNOISE, SNRWM = SWM/SDNOISE, SNRLESION = SLESION/SDNOISE

^b^Data are expressed as mean and SD or median and interquartile rage, as appropriate

There was no need to repeat the sequence for movement, and no periprocedural complications were recorded. The duration of the magnetic resonance imaging was 30 min (SD ± 6.39 min).

**Fig. 3:**
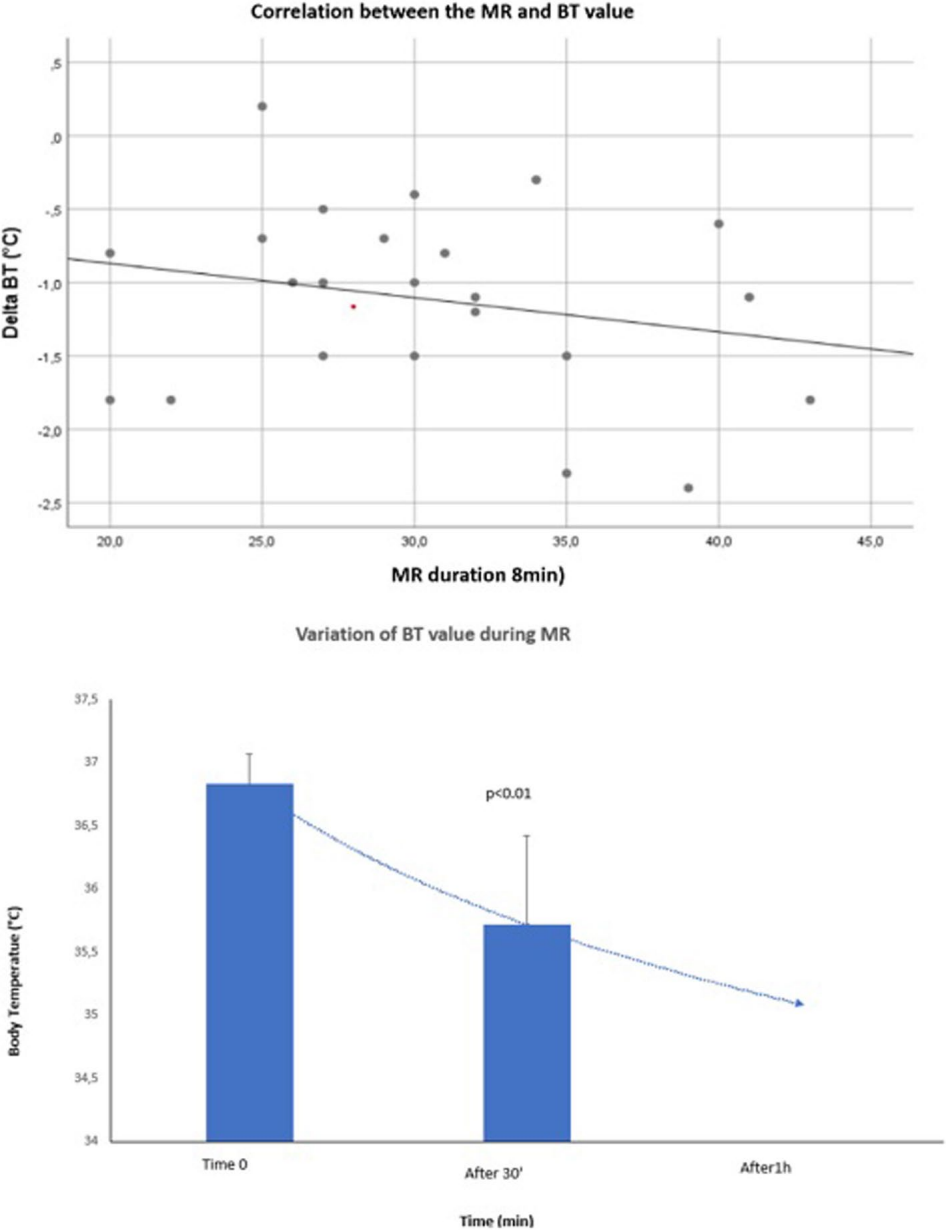
Correlation between MR duration and body temperature. MR: magnetic resonance; BT: body temperature

During the radiological procedure, we observed a statistically significant reduction in rectal temperature value between the start and the end of the procedure: the median rectal temperature value was 36.8 °C (36.7–36.9) at the beginning of the MR and 35.8 °C (35.3–36.2) at the end of MR (*p* < 0.01), with a Delta reduction of BT value of − 1 °C (− 1.5 to − 0.7) and a Pearson correlation coefficient: − 0.23, the coefficient *R*^2 of 0.054 (Fig. 3). The average time to restart full enteral feeding was 1.63 ± 0.83 h.

## Discussion

Prematurity is considered one of the most significant risk factors for emergency intubation during MR sedation in children. This vulnerability may be attributed to the immaturity of respiratory centers and the increased laxity of the upper airway wall [20]. These characteristics explain the heightened susceptibility of preterm infants to oxygen desaturations during sedation for MR examinations. In their study, Havidich et al. highlighted that preterm and former preterm infants undergoing sedation anesthesia for procedures outside the operating room should be considered a risk group for developing adverse events [4]. The author lists in their study that mechanisms implicated in the development of apnea and respiratory abnormalities also include altered carotid chemoreceptor responses to hypoxia and hyperoxia, various neurotransmitters, genetic predisposition, and laryngeal chemoreflexes.

Several reports have attempted to address this issue by improving anesthesia-sparing techniques, even if it might lead to a lower quality of imaging or necessitate rescheduling new MR appointments [21, 22]. Our findings suggest that lying in a lateral position during sedation could reduce respiratory adverse events compared to literature data [2]: oxygen desaturation and apnea episodes occurred in only one (4.3%) and two (8.6%) patients respectively, accounting for an incidence of adverse events in 2/23 (9%) newborns. There is no evidence in the literature suggesting that particular body positions during spontaneous breathing of the neonate are effective in producing sustained and clinically relevant improvement [23]. However, there are some physiological findings in older sedated children about upper airway patency in the lateral position [24]. It is unclear whether effects depend on apneic etiology (central, obstructive, or mixed apneic episodes), but we suppose that preterm infants are exposed to a high rate of apnea due to occlusion events. Lateral lying could alleviate airway occlusion and may decrease the activity of excited sympathetic nerves [25].

Our procedure offered a good quality of imaging and satisfaction of the operators. Subjective and objective radiologic analyses appeared diagnostically equivalent to those typically obtained in the supine position. Scores assigned in all subcategories during the subjective assessment of images by both neuroradiologists were not affected by position. Despite a subjective score being common in this age class for image quality assessment [26], we considered an objective evaluation too, with an indexed analysis on the reduced number of excitation and weight patients. In our sample, the values of SNRGM, SNRWM, and CNRGM were consistent with the expected values and those observed in images acquired from patients in the supine position [27–29].

The positive respiratory outcome offers a silver lining for overall preterm homeostasis. As suggested by the literature, shortening the examination time leads to an improvement in the patient’s clinical stability [30]. Repeated acquisition, discontinuation due to apnea events, and major adverse events expose these little children to additional anesthesia time in an uncomfortable environment.

Our results show that even brief procedures are associated with a decrease in body temperature confirming the difficulty of keeping warm so small patients. During the radiological procedure, we observed a statistically significant reduction in rectal temperature value between the start and the end of the procedure (*p* < 0.01). There was a negligible negative relationship between the MR duration and rectal temperature value.

Preterm infants are at higher risk for hypothermia than term infants due to their lower amount of subcutaneous fat, less insulating skin, lower stores of brown fat and glycogen, and poor vasomotor control [31]. In the case of hypothermia, the risk of desaturation, respiratory failure, and metabolic complications, such as hypoglycemia and poor food tolerance, increases accordingly [32]. We can speculate that sedation in the lateral position would improve respiratory activity, leading to a direct reduction in respiratory effort and energy consumption. Additionally, the low rate of adverse events and number of repeated imaging should indirectly affect the timing of MR and, consequently, the risk of hypothermia, allowing for faster recovery and resumption of feeding. Furthermore, it is reasonable to believe that more recent advances in fasting regimens could be helpful in maintaining metabolic wellness [33]. While metabolic homeostasis takes several factors into account, limiting changes in standard intake (reduced fasting and rapid resumption) should be considered a crucial clinical condition.

Actually, we found no evidence of food intolerance upon return to the neonatal ward after the examination, and the time required to achieve complete enteral nutrition was consistent with normal clinical practice.

In the entire sample included in the study, the MR examination was carried out without interruptions. This is an important aspect as it allowed for a reduction of sevoflurane environmental diffusion. Open circuits in spontaneous breathing do not allow the scavenging of halogenates, and professional exposition and their pollution effect in terms of global warming potential should be always kept in mind [34].

An important limitation of the study is the lack of randomization with supine patients. The original design was indeed a randomized controlled trial but local IRB refused it, due to positive data expressed in a previous experience [35]. Future high-quality cross-over or randomized studies are advisable for more accurate comparisons of different patient positions on MR scanning beds and alternative sedation techniques, like alpha-2-agonists. These agents result in a lower incidence of respiratory depression, while still having a greater hemodynamic impact. However, they may be less advantageous in terms of the time required to achieve an adequate level of sedation and patient immobility during the procedure [21, 36]. Furthermore, a comparison of our first-choice technique with sevoflurane sedation and another pharmacological and non-pharmacological approach would be interesting in determining a clear risk–benefit balance.

Our experience, although with a single-center limited sample, suggests that lateral position for sedation of preterm infants scheduled for MR examination is a reliable option. Patient safety andquality of imaging analysis should be confirmed by further rigorous trials to verify if the process of care for the patients is adequate for their needs.

## Data Availability

No datasets were generated or analysed during the current study.
